# Measured Effects of Wnt3a on Proliferation of HEK293T Cells Depend on the Applied Assay

**DOI:** 10.1155/2015/928502

**Published:** 2015-12-22

**Authors:** Patricia Reischmann, Johanna Fiebeck, Nadine von der Weiden, Oliver Müller

**Affiliations:** ^1^University of Applied Sciences Kaiserslautern, Molecular Biology, Amerikastraße 1, 66482 Zweibrücken, Germany; ^2^PHAST GmbH, Entenmühlstraße 48, 66424 Homburg, Germany

## Abstract

The Wnt signaling pathway has been associated with many essential cell processes. This study aims to examine the effects of Wnt signaling on proliferation of cultured HEK293T cells. Cells were incubated with Wnt3a, and the activation of the Wnt pathway was followed by analysis of the level of the *β*-catenin protein and of the expression levels of the target genes* MYC* and* CCND1*. The level of *β*-catenin protein increased up to fourfold. While the mRNA levels of c-Myc and cyclin D1 increased slightly, the protein levels increased up to a factor of 1.5. Remarkably, MTT and BrdU assays showed different results when measuring the proliferation rate of Wnt3a stimulated HEK293T cells. In the BrdU assays an increase of the proliferation rate could be detected, which correlated to the applied Wnt3a concentration. Oppositely, this correlation could not be shown in the MTT assays. The MTT results, which are based on the mitochondrial activity, were confirmed by analysis of the succinate dehydrogenase complex by immunofluorescence and by western blotting. Taken together, our study shows that Wnt3a activates proliferation of HEK293 cells. These effects can be detected by measuring DNA synthesis rather than by measuring changes of mitochondrial activity.

## 1. Introduction

The Wnt signaling pathway is known to play a key role in regulating cellular differentiation, proliferation, survival, and apoptosis [1–3]. Wnt proteins are a highly conserved family of secreted lipid-modified cysteine-rich glycoproteins [4] that activate signaling cascades inside the cell by binding to a member of the Frizzled (Fz) family of G-protein-coupled receptors on the extracellular matrix [5]. There are 19 members of vertebrate Wnts [6], which can be grouped into two main classes based on their ability to stabilize cytosolic *β*-catenin: canonical and noncanonical pathway [7–9]. Recent works have also shown that certain Wnt ligands, like Wnt5a, are able to activate more than only one Wnt signaling pathway [10, 11], so the strictly binary classification is becoming more and more outdated. Malfunctioning of Wnt signaling pathway is related to several diseases like osteoporosis [12], Crohn's disease [13], Alzheimer's disease [14], schizophrenia [15], and especially cancer [1, 16].

The ligand Wnt3a belongs to *β*-catenin dependent (canonical) Wnt signaling, which binds to frizzled transmembrane receptors. Subsequently the low-density lipoprotein receptor-related protein 5 (LRP5) or LRP6 receptor complex activates cytoplasmic disheveled proteins, which trigger the inhibition of glycogen synthase kinase 3*β* (GSK-3*β*). The protooncoprotein *β*-catenin accumulates in the cytoplasm as a consequence of the disassembly of the destruction complex that is formed by adenomatosis polyposis coli (APC), AXIN, and GSK-3*β*. Thus, *β*-catenin can be translocated into the nucleus where it activates the transcription of target genes mediated by T-cell specific transcription factor (TCF)/lymphoid-enhancing factor (LEF) [17]. These genes are responsible to regulate essential physiological processes in embryonic and adult development such as cell proliferation, differentiation, morphogenesis, and cell adhesion [18].

With the indication of proliferation, there is a complex interplay between the canonical Wnt signaling and the cell cycle. The Wnt/*β*-catenin signaling pathway is significant in stimulating progression of G1-phase by inhibiting GSK-3*β*, which regulates cell cycle effectors and growth regulators [19].

One direct target gene of the Wnt/*β*-catenin signaling pathway is the protooncogene* MYC*, which was identified as a Wnt target in colon cancer cells [20]. The transcription regulator c-Myc controls many cell functions, especially the regulation of cell cycle progression [21]. The cyclin D1 coding gene* CCND1* is also a target gene of the Wnt/*β*-catenin pathway, which was described in colon cancer cells [22, 23]. Cyclin D1 as an activator of cyclin dependent kinase (cdk) 4 or cdk 6 drives the G1/S phase transition [24, 25].

There are several ways to measure Wnt3a induced cell proliferation. One nonradioactive method is an immunoassay based on incorporation of 5-bromo-2′-deoxyuridine (BrdU) into DNA to determine DNA synthesis, which correlates to S phase transition and thus to cell proliferation [26]. Another method uses the measurement of mitochondrial activity of viable cells. In the MTT assay an insoluble blue formazan product is produced by mitochondrial dehydrogenases by reduction of MTT (3-(4,5-dimethylthiazol-2-yl)-2,5-diphenyl tetrazolium bromide) [27].

In this study we investigated the proliferation of HEK293T cells under the influence of Wnt3a ligands by using the MTT as well as the BrdU assay. Furthermore we compare the results from these assays with mitochondrial activity of Wnt3a treated cells as measured by immunostaining of the electron transport chain protein SDHA (succinate dehydrogenase complex subunit A).

## 2. Materials and Methods

### 2.1. Cells and Cell Cultivation

The human embryonal kidney cells, HEK293T, were cultured in Dulbecco's modified Eagle's medium (DMEM) supplemented with 10% fetal bovine serum (PAN Biotech, Aidenbach, Germany), 1% nonessential amino acids, and 1% penicillin/streptomycin (Sigma Aldrich, St. Louis, USA) at 37°C in 5% CO_2_.

### 2.2. Proliferation Assays

MTT and BrdU assays were performed on 96-well plates. Ten thousand cells were seeded per well and cells were stimulated with escalating doses of Wnt3a (R&D Systems, Minneapolis, USA), namely, 0, 10, 50, 100, 150, and 200 ng/mL, for 24, 48, and 72 h. MTT (Sigma-Aldrich, St. Louis, USA) was dissolved in PBS at 5 mg/mL and 20 *μ*L of MTT solution was added to each well followed by an incubation of three hours at 37°C in 5% CO_2_. Medium with MTT was then flicked out and 200 *μ*L DMSO was added. To dissolve the crystals, cells were shaken for 15 min at room temperature. Plates were read out by an ELISA reader (Tecan Group Ltd., Männedorf, Switzerland) at 590 nm. For BrdU assay a commercial kit “Cell Proliferation ELISA BrdU colorimetric” (Roche, Mannheim, Germany) was used according to the manufacturer's instructions. After addition of BrdU labelling solution the cells were incubated for three hours at 37°C in 5% CO_2_. After the final washing step 100 *μ*L/well substrate solution was added to the cells and the reaction was stopped after 15 min incubation at room temperature by using sulphuric acid. Plates were read out immediately by an ELISA reader at 450 nm. All assays were done in triplicate (*n* = 12). For calibration cells were seeded in different numbers (0,1 × 10^4^ to 3 × 10^4^).

### 2.3. Immunocytochemistry and Fluorescence Microscopy

HEK293T cells were seeded into 24-well plates and treated with 0–200 ng/mL Wnt3a at a confluency of 30% and fixed in 4% formaldehyde after 24, 48, and 72 h. SDHA antibody (Abcam, Cambridge, UK) at a dilution of 1 : 200 was added and cells were incubated for one hour. Cell nuclei were counterstained with DAPI for 5 min. Cells were analyzed via fluorescence microscopy (Observer. Z1 from Zeiss, Jena, Germany) and the accompanying software AxioVision 4.7.

### 2.4. Protein Gel Electrophoresis and Western Blot

Using the Bradford-Assay protein concentrations in whole-cell lysates were determined. Equal protein amounts were loaded on SDS-polyacrylamide gels (NuPAGE, 4–12%, Life Technologies, Carlsbad, USA). Gels were run at 150 V for 1 h and transferred onto nitrocellulose membranes via the iBlot System (Life Technologies). Membranes were analysed using primary antibodies against SDHA, *β*-catenin, c-Myc, Cyclin D1, and *β*-actin (Cell Signaling, Cambridge, UK), diluted 1 : 1000 in TBS-Tween with 3% milk, followed by incubation with an appropriate horseradish peroxidase-conjugated secondary antibody (dilution 1 : 2000). Protein bands were visualized using ECL detection.

### 2.5. RNA Isolation and RT-PCR

Total RNA was isolated using NucleoSpin RNA II (Macherey-Nagel, Düren, Germany) and 1 *μ*g RNA was reverse-transcribed with Verso cDNA-Kit (Thermo Fisher Scientific, Schwerte, Germany) in a volume of 40 *μ*L. Sequences of the primers (MWG, Munich, Germany) used for semiquantitative reverse transcriptase PCR are for* GAPDH* 5′-catggtgctgagatttgccaac-3′ (forward) and 5′-tcaacaccttgaccttctcatcac-3′ (reverse), for* MYC* 5′-ccgagcaaggacgcgactctc-3′ (forward) and 5′-gcctttcagagaagcgggtcct-3′ (reverse), and for CCND1 5′-gcctgaacctgaggagcccca-3′ (forward) and 5′-gtcacacttgatcactctgg-3′ (reverse). PCR amplification was performed using My Taq Red Mix (Bioline, Luckenwalde, Germany) according to the manufacturer's protocol. The reaction was performed with preliminary denaturation for 2 min at 94°C, followed by 30 cycles of denaturation at 94°C for 1 min, annealing at 55°C (*CCND1*), 57°C (*GAPDH*), or 60°C (*MYC*) for 30 sec, and extension at 72°C for 1 min. Final extension step lasted 10 min at 72°C. PCR products were analysed by electrophoresis on a 1,5% agarose gel.

### 2.6. Data Analysis

Microsoft Excel software was used for data management, including calculating standard deviations. western blots and agarose gels were scanned by gel documentation system Quantum ST4 (Vilber Lourmat, Eberhardzell, Germany). The bands were quantified by using ImageJ software (Wayne Rasband, NIH, Bethesda, USA).

## 3. Results

### 3.1. Effects of Wnt3a on Induction of *β*-Catenin and Typical Wnt Target Genes

**Figure 1:**
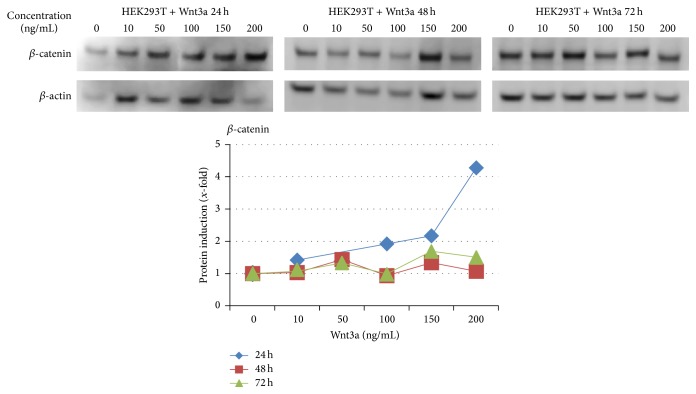
Western blot analysis of HEK293T cells treated with 0–200 ng/mL Wnt3a for 24, 48, or 72 h. *β*-catenin slightly increased 24 hours after treatment. *β*-actin antibody was used as loading control. For quantification, the protein levels were normalized to the level of *β*-actin and the untreated control was set to 1.

**Figure 2:**
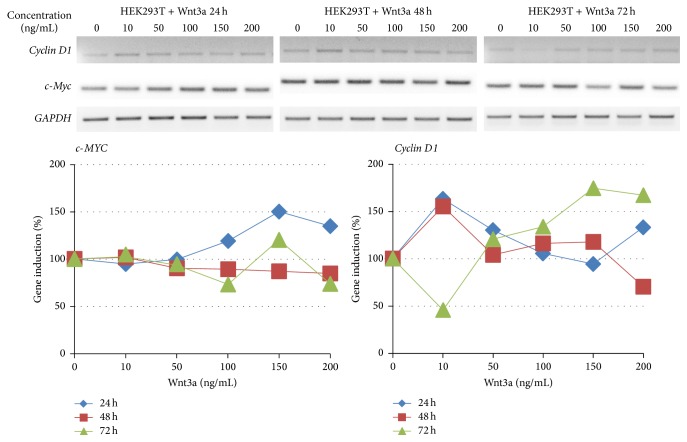
Results of RT-PCR. Exponentially growing HEK293T cells were treated with 0–200 ng/mL Wnt3a for 24, 48, or 72 h. After these time points total RNA was isolated and semiquantitative RT-PCR was performed using* CCND1*,* MYC,* and, as loading control,* GAPDH* specific primers. For quantification, the expression was normalized to the level of* GAPDH* expression and the untreated control was set to 100%.

**Figure 3:**
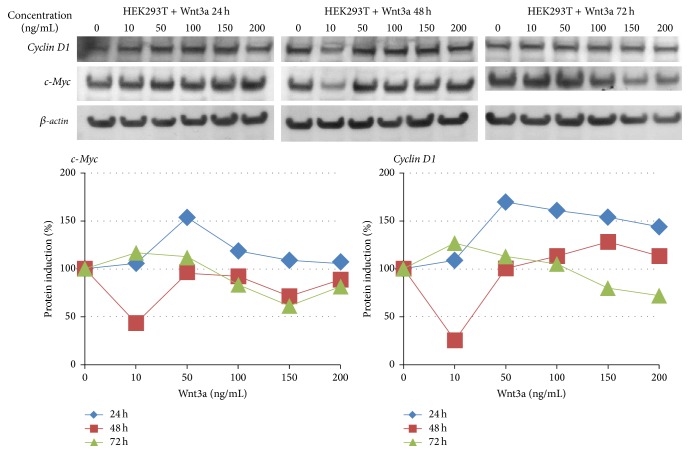
Western blot analysis of HEK293T cells treated with 0–200 ng/mL Wnt3a for 24, 48, or 72 h. The membrane was incubated with cyclin D1 and c-Myc specific antibodies. *β*-actin antibody was used as loading control. For quantification, the protein levels were normalized to the level of *β*-actin and the untreated control was set to 100%.

In order to prove activation of the Wnt pathway, we detected the amount of *β*-catenin protein concentration in HEK293T cells after treatment with Wnt3a (Figure 1). After 24 hours of treatment, *β*-catenin protein concentration rose with increasing concentrations of Wnt3a. The highest *β*-catenin protein concentration was identified at 24 hours with a concentration of 200 ng/mL Wnt3a. Here the quantification showed a fourfold increase compared to the control. At time points 48 and 72 hours after treatment the effect of *β*-catenin induction is not clearly recognizable any longer. Furthermore we analyzed the regulation of the Wnt target genes* CCND1* and* MYC* after treatment with Wnt3a both by semiquantitative RT-PCR and by western blot (Figures 2 and 3). A basal level of c-Myc and cyclin D1 was detected in untreated cells. The results of RT-PCR showed marginal changes in the amount of* MYC* cDNA and an induction of CCND1 after 24 and 48 hours with 10 ng/mL Wnt3a, as well as after 72 h with a concentration of 100–200 ng/mL Wnt3a. A slightly increased protein level of c-Myc was observed with a concentration of 50 ng/mL after 24 hours. The western blot quantification of cyclin D1 showed an increase by a factor of 1,5 with a Wnt3a concentration from 50 to 200 ng/mL after 24 hours. The protein level of c-Myc ascended in the same way with the concentration of 50 ng/mL Wnt3a after 24 hours.

### 3.2. Cell Proliferation Assays

**Figure 4:**
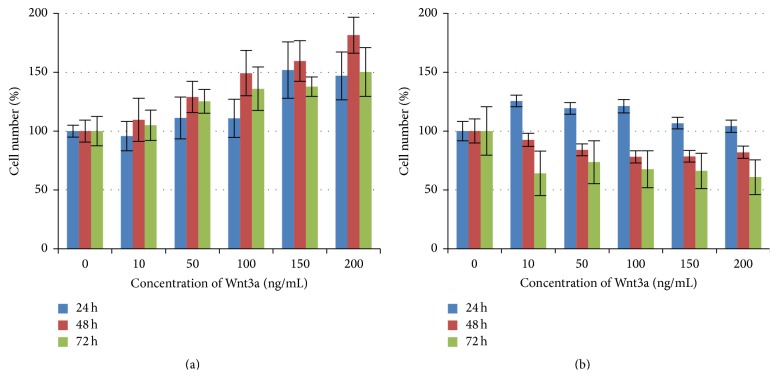
Results of the proliferation assays. HEK293T cells were treated with different concentrations of Wnt3a. The values of the assays were measured after 24 h, 48 h, and 72 h. Bars are showing the cell number per well expressed as a percentage in comparison to the untreated control (vertical axis), which was normalized to 100%. All assays were made in triplicate. (a) BrdU assay. (b) MTT assay.

To study the effects of Wnt proteins on cell growth, HEK293T cells were grown in 96-well plates for 24 hours before they were treated with Wnt3a protein concentrations of 0, 10, 50, 100, 150, or 200 ng/mL, respectively. After 24, 48, and 72 hours of incubation, MTT and BrdU assays were performed. The BrdU assays show a clear trend; with increasing concentrations of Wnt3a the associated read-out increased (Figure 4(a)), in which Wnt3a indicates the highest growth rate after 48 hours and a concentration of 200 ng/mL.

In contrast to the BrdU assays, the MTT assays did not show a correlation between Wnt concentrations and cell numbers. Cell numbers stayed all about the same or decline in comparison to their relevant controls (see Figure 4(b)).

### 3.3. Mitochondrial Activity

**Figure 5:**
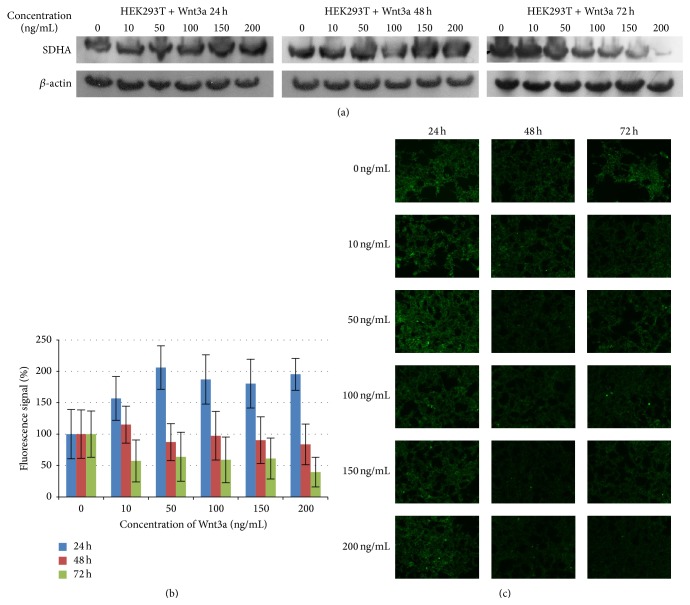
Mitochondrial activity of Wnt3a treated HEK293T cells. (a) Exponentially growing HEK293T cells were exposed to different Wnt3a concentrations for the indicated times. Protein extracts were prepared and used for western blot analysis. The membrane fractions were incubated with antibody against SDHA or, as loading control, against *β*-actin. (b and c) HEK293T cells were subjected to immunocytochemical analysis. SDHA protein was detected by green fluorescence via fluorescence microscopy at 200x magnification. Bars are showing band intensity expressed as percentage in comparison to the untreated control (vertical axis), which was normalized to 100%.

To further substantiate the role of Wnt3a relating to the mitochondrial activity in HEK293T cells, amounts of SDHA protein were determined by western blot as well as by immunocytochemical analysis. Succinate dehydrogenase (SDH) with its subunit SDHA is a key component of the citric acid cycle, which is involved in complex II of the mitochondrial electron transport chain and is responsible for transferring electrons from succinate to ubiquinone. Cells were treated equally to proliferation analysis experiments and incubated with SDHA antibody as described above. The results (Figure 5) showed an increasing fluorescence signal and also a more intense signal at western blot relative to the control after 24 hours of Wnt3a treatment. After 48 hours the protein level registered by immunofluorescence microscopy or by western blot analysis showed no significant change. After 72 hours of Wnt3a treatment even a decreasing signal was observed in both experiments. The highest signal was measured after 24 hours with 50 ng/mL Wnt3a.

## 4. Discussion

Wnt proteins are involved in various cellular functions including the regulation of gene expression for cell cycle and proliferation [28]. Wnt3a is known as a promoter of proliferation and migration of human lens epithelial cells [29], also for proliferation of fibroblasts [30] or as a regulator of proliferation of pancreatic NIT-1 beta cells [31].* MYC* and* CCND1* are target genes of the Wnt signaling pathway in mammalian cells. For example, Yoon et al. exposed that Wnt signaling activates mitochondrial biogenesis by performing a large-scale RNAi screen; they identified genes which affect mitochondrial function, amongst other* MYC* [32].

In this work we determined the effects of Wnt3a on proliferation of HEK293T cells. For this we applied two major assays, which are widely used for analysis of proliferation rates of cultured cells, namely, the MTT and the BrdU assays. In doing so, the specific effects of Wnt ligands on DNA synthesis and mitochondrial activity were detected and compared directly.

In order to establish our system and to prove the effect of Wnt3a on the Wnt pathway in HEK293T cells we demonstrated the induction of *β*-catenin after 24 hours of Wnt3a treatment and we analyzed the expression levels of* MYC* and* CCND1*. The level of both target genes also increased after 24 hours of Wnt3a exposition. C-Myc has a key role in G1-phase progression; it upregulates cyclin D1 [33] and represses p21 and p27 [34, 35]. Cyclin D1 in turn promotes phosphorylation and inhibition of the retinoblastoma (Rb) complex, which itself upregulates the cyclin E level [19, 36]. The enrichment of cyclin E leads to the transition of the G1/S-phase checkpoint [37].

During G1-phase mitochondria have to supply a high amount of energy [38]. This oxidative phase is followed by a reductive period during S-/G2-/M-phase of the cell cycle, in which replication of DNA and proliferation of mitochondria take place [39].

Proliferation rates of Wnt3a treated HEK293T were different, when measured by the two assays used in this study. We explain the difference by the dissimilar detection mechanisms of these methods. The MTT assay measures the activity of mitochondria; on the other hand the BrdU assay detects the relative amount of newly synthesized DNA. Wagner et al. refer to a highly significant relationship between BrdU and MTT in their results with canine lymphocyte proliferation, although the BrdU assay is proved to be more sensitive than the MTT assay [40]. The apparently negative effect of Wnt3a on cell numbers could be explained by a decline in mitochondrial activity. The BrdU assay, however, showed a promoting effect on DNA synthesis in these cells. The experiments of mitochondrial activity showed that only after 24 hours of Wnt3a treatment the SDHA level was increased. The activation with recombinant Wnt3a has probably no effect on the mitochondrial activity after 48 hours or later. According to this time-limited effect is the decreasing signal during MTT assay after 48 and 72 hours. This is also confirmed by the declining accumulation of *β*-catenin by Wnt3a after 24 hours. Other publications support this assumption that Wnt3a treated cells indicate an increased *β*-catenin level only during a 24-hour period [41, 42]. We explain our results, which are contrary to the published proliferating effect of Wnt3a by the fact that most studies used Wnt3a overexpressed cells, Wnt3a-conditioned medium, or serum-starved cells with recombinant Wnt protein for activation Wnt/*β*-actin signaling pathway.

In conclusion, results from BrdU and the MTT assays are hardly comparable when measuring the effects of Wnt3a on the proliferation rate of HEK293T cells. Differences can be explained by their different points of application, namely, the mitochondrial activity and the synthesis of DNA. In order to give definitive evidence about the cellular proliferation rate, interpretation of outcomes of one single method might be misleading. For evaluation of cell proliferation the direct counting of cells by optical methods would mirror best the real situation. It would be interesting to compare the corresponding outcomes with the results of biochemical assays like BrdU and MTT.
